# Progresses in overcoming the limitations of in vitro erythropoiesis using human induced pluripotent stem cells

**DOI:** 10.1186/s13287-024-03754-9

**Published:** 2024-05-15

**Authors:** Hyeonwoo Ju, Yeowon Sohn, Yoojun Nam, Yeri Alice Rim

**Affiliations:** 1 Department of Biotechnology, Yonsei University, Seoul, 03722 Korea; 2 Department of Biohealth Regulatory Science, Sungkyunkwan University, Suwon, South Korea; 3 YiPSCELL Inc., L2 Omnibus Park, Banpo-dearo 222, Seocho-gu, Seoul, 06591 Republic of Korea; 4 CiSTEM laboratory, Convergent Research Consortium for Immunologic Disease, Seoul St. Mary’s Hospital, College of Medicine, The Catholic University of Korea, Seoul, 06591 Republic of Korea

**Keywords:** Induced pluripotent stem cell, Red blood cell, Erythropoiesis, Enucleation, Bioreactors

## Abstract

Researchers have attempted to generate transfusable oxygen carriers to mitigate RBC supply shortages. In vitro generation of RBCs using stem cells such as hematopoietic stem and progenitor cells (HSPCs), embryonic stem cells (ESCs), and induced pluripotent stem cells (iPSCs) has shown promise. Specifically, the limited supplies of HSPCs and ethical issues with ESCs make iPSCs the most promising candidate for in vitro RBC generation. However, researchers have encountered some major challenges when using iPSCs to produce transfusable RBC products, such as enucleation and RBC maturation. In addition, it has proven difficult to manufacture these products on a large scale. In this review, we provide a brief overview of erythropoiesis and examine endeavors to recapitulate erythropoiesis in vitro using various cell sources. Furthermore, we explore the current obstacles and potential solutions aimed at enabling the large-scale production of transfusable RBCs in vitro.

## Introduction

Since the introduction of induced pluripotent stem cells (iPSCs) in 2006 [1], various attempts have been made to utilize them in clinical applications [2] owing to their pluripotency and self-renewal capacity [3]. Alternative transfusable oxygen carriers are highly desirable for addressing scarcities in blood products. An imminent red blood cell (RBC) supply shortage is highly probable not only because of low birth rates and an aging society [4, 5], but also because of pandemic-induced low blood donation rates [6, 7].

Numerous trials have been conducted on the development of artificial blood or blood substitutes in an effort to create a diversified blood supply chain, including artificial oxygen carriers (AOCs), hemoglobin-based oxygen carriers (HBOCs), and perfluorocarbon-based oxygen carriers (PFCOCs) [8–11]. These noncellular products have critical hurdles to overcome, including biocompatibility issues and side effects such as vasoconstriction, which eventually led to failure in clinical trials [8, 12, 13]. Since the 20th century, research on generating RBCs has been published [14]. Various cell sources have been used to generate RBCs, including hematopoietic stem and progenitor cells (HSPCs) [15], embryonic stem cells (ESCs) [16], and iPSCs [17]. Recently, erythropoiesis using iPSCs has become more common due to the limited availability of HSPCs and ethical considerations for ESCs, which prevent large-scale RBC production [18]. Compared to other cell sources, iPSCs can be generated from somatic cells, are an unlimited source of cells, and can differentiate into hematopoietic lineages, making them an alternative for HSPCs [19]. Although iPSCs are a good cell source for in vitro erythropoiesis, some difficulties remain to be overcome, such as enucleation in the terminal maturation phase, immature RBCs, and challenges in large-scale manufacturing. These obstacles hinder erythropoiesis from being used to manufacture transfusable blood products. In this review, we first briefly explain the fundamental principles of erythropoiesis and focus on the in vitro erythropoiesis of iPSCs. We will then review previous attempts at in vitro RBC generation using various cell sources and the limitations of in vitro erythropoiesis. Furthermore, we discuss possible breakthroughs that may provide solutions to these limitations.

## In vivo and in vitro erythropoiesis

Erythropoiesis is the process of creating mature RBCs from hematopoietic stem cells and consists of multiple steps involving erythroid progenitor cells, erythroblast precursor cells, reticulocytes, and erythrocytes [20–22]. There are two types of erythropoiesis: primitive and definitive erythropoiesis [23]. Primitive erythropoiesis occurs in the yolk sac and in the early stages of embryonic development, and involves the initial generation of erythroid lineage cells throughout the human lifespan [24]. Unlike primitive erythropoiesis, definitive erythropoiesis first occurs in the fetal liver, then migrates to the spleen, and finally to the bone marrow shortly before birth [23]. Primitive erythropoiesis ceases at the end of the embryonic stage, thereby generating erythroblasts that differentiate into primitive erythrocytes. Definitive erythropoiesis occurs later in the embryonic stage compared to primitive erythropoiesis, and also occurs throughout the lifespan [23].

### In vivo erythropoiesis

In vivo erythropoiesis can be subdivided into multiple stages [25]. First, hematopoietic stem cells (HSCs) differentiate into multipotent progenitors (MPPs), which later differentiate into specific erythroid megakaryocyte-primed multipotent progenitors (EMkMPPs) [26, 27]. EMkMPPs then undergo a commitment phase, lose their multipotency, differentiate into burst-forming unit erythroid (BFU-E), and enter the erythroid lineage [28]. BFU-E further differentiates into colony-forming unit erythroid (CFU-E); BFU-E and CFU-E are erythroid progenitors [29].

CFU-E cells later generate erythroid precursor cells by differentiating into proerythroblasts (Pro-E), basophilic erythroblasts (Baso-E), polychromatic erythroblasts (Poly-E), and orthochromatic erythroblasts (Ortho-E) [22]. After undergoing further differentiation from Pro-E, the chromatin of the cell condenses and moves to the pole (nuclear condensation and polarization) [30, 31], which are prerequisites for enucleation. The cells also accumulate hemoglobin during these phases. In Ortho-E, the nucleus undergoes pyknosis and becomes rich in hemoglobin [21]. Ortho-E then expels its nucleus and becomes a reticulocyte. The expanded nuclei become pyrenocytes, which are later phagocytosed by macrophages [32]. Finally, the reticulocytes differentiate into terminally mature erythrocytes [33].

In vivo definitive erythropoiesis is driven by specific niches in the bone marrow called erythroblastic islands (EBIs) [34]. EBIs are composed of central macrophages and several surrounding erythroblasts [34, 35]. EBIs are crucial for the development of mature RBCs, in which central macrophages function in various ways to help erythroblasts undergo terminal maturation [35]. First, central macrophages secrete cytokines that promote erythroblast differentiation. These macrophages express insulin-like growth factor 1 (IGF1), IL-18, and vascular endothelial growth factor B (VEGF-B); IGF1 is reported to promote erythropoiesis in adjacent erythroblasts, whereas the functions of IL-18 and VEGF-B require further research [36, 37]. These macrophages also anchor to surrounding erythroblasts by expressing adhesion molecule ɑV integrin, which binds to intracellular adhesion molecule 4 (ICAM4) located on the surface of erythroblasts, thereby providing EBI integrity [36, 38]. They also supply iron to erythroblasts, which is essential for the production of heme, the main constituent of hemoglobin [39]. Since the main function of macrophages in vivo is to phagocytize debris, they also remove byproducts released by erythroblasts during erythroid maturation [40]. Pyrenocytes (which are extruded nuclei from Ortho-E), organelles disposed of by reticulocytes, and RBCs whose lifespan has been reached, are all phagocytized by these macrophages [41]. In addition, heme is recycled from aged RBCs by macrophages and returned to new hemoglobin-synthesizing erythroblasts [42].

## Previous attempts of in vitro erythropoiesis

There have been many attempts to generate erythropoiesis in vitro to produce artificial blood products, since the prospects of other blood substitutes such as HBOCs and PFCOCs have not been promising. In the following section, we will summarize some important milestones in in vitro erythropoiesis, in chronological order.

The first attempt at in vitro erythropoiesis was attempted by the Douay group in 2002 and used CD34^+^ HSPCs from bone marrow, cord blood, and peripheral blood. They generated a pure erythroid precursor population which was amplified 200,000-fold from CD34^+^ hematopoietic stem cells obtained from human cord blood; when these erythroid populations were transfused into NOD/SCID mice, they achieved a low enucleation rate of 4% in vivo [15]. The Douay group next developed a three-step protocol using coculture with either murine MS-5 stromal cells or human mesenchymal cells, which also led to a 200,000-fold amplification of CD34^+^ HSCs, with an enucleation rate of up to 91% [43]. Miharada et al. built on the Douay group’s achievement of generating enucleated RBCs in the presence of feeder cells, and reported a four-step protocol for producing enucleated RBCs using CD34^+^ HSCs from cord blood without feeder cells, in media containing stem cell factor (SCF), erythropoietin (EPO), interleukin-3 (IL-3), vascular endothelial growth factor (VEGF), and insulin-like growth factor-II (IGF-II). They achieved a near 80% enucleation rate [44]. Fujimi et al. generated RBCs with an enucleation rate of 99.4% from cord blood CD34^+^ HSCs via a four-step protocol, using medium with SCF, Flt-3/Flk-2 ligand, thrombopoietin (TPO), and EPO. They co-cultured erythroblasts with CD34^+^ HSC-differentiated macrophages from a different donor, successfully resembling EBI in ex vivo conditions [45]. Timmins et al. proposed protocols for manufacturing ultrahigh-yield RBCs from cord blood CD34^+^ HSCs using agitating bioreactors [46]. They were able to produce over 500 units of RBCs from a single umbilical cord blood donation, with over 10^8^-fold expansion using a fully defined culture medium, ultimately achieving an enucleation rate of over 90%. Around the same time, Douay et al. transfused cultured RBCs (cRBCs) into humans, which were differentiated from peripheral blood CD34^+^ HSCs with improved RBC characteristics, including deformability, enzyme content, capacity of their hemoglobin as an oxygen carrier, and expression of blood group antigens [47]. The transfused RBCs had a half-life comparable to that of native RBCs (28 ± 2 days). The methods used in these two studies hold promise for overcoming critical obstacles such as the low enucleation rate and immaturity of cRBCs, and facilitate the transition to utilizing ex vivo-generated RBCs in human clinical transfusions [46, 47].

Attempts have been made to generate RBCs in vitro using PSCs. Early in the 21st century, Kaufman et al. reported that ESCs have potential as an alternative source of HSCs, as ESCs cocultured with murine bone marrow S17 cells or yolk sac endothelial C166 cells differentiated into CD34-expressing HSPCs [48]. A similar study was conducted by Vodyanik et al., in which ESCs differentiated into high-purity CD34^+^ HSCs when cocultured with murine bone marrow OP9 cells, and these CD34^+^ cells were able to differentiate into erythroid, lymphoid, and myeloid lineage cells [49]. Olivier et al. modified Douay et al.’s protocols, which were originally created to generate enucleated RBCs from cord blood or peripheral blood CD34^+^ HSCs [15, 43], to enable large-scale RBC production from human ESCs (hESCs) [16]. They developed a five-step protocol to generate ex vivo cRBCs, and co-cultured hESCs with fetal human liver clone B (FH-B-hTERT) cells to differentiate them into CD34^+^ HSCs. These were then co-cultured with murine bone marrow MS5 cells to promote terminal maturation of the erythroid lineage, resulting in over a 5000-fold increase expansion. However, enucleated cells were not reported. Ma et al. similarly developed a process in which hESC-derived enucleated RBCs were prepared via co-culture with mouse FL-derived stromal cells (mFLSCs), and these cultured RBCs demonstrated maturity comparable to that of native RBCs. The cRBCs had low ε-globin expression and high β-globin expression, indicating definitive erythropoiesis [50, 51].

Since the first development of iPSCs in 2006 [1], their potential as precursors to RBCs in vitro has been explored. Hanna et al. were the first to report the potential of iPSCs to differentiate into RBCs [52]. They were able to differentiate iPSCs by editing the sickle hemoglobin allele into hematopoietic progenitors in vitro, and when these progenitor cells were transplanted into mice, they differentiated into RBCs with proper morphology. Schenke-Layland et al. and Choi et al. reported that murine iPSCs differentiated into murine hematopoietic lineages while human iPSCs differentiated into human hematopoietic lineages. These findings show that iPSCs have the potential for erythroid differentiation [53, 54]. Douay et al. published the first report on the complete ex vivo differentiation of hiPSCs into RBCs, using SCF, FLT-3 ligand, TPO, bone morphogenetic protein 4 (BMP4), VEGF, IL-3, IL-6, and EPO for the formation of human embryoid bodies (hEBs) in the first step. They then used SCF, EPO, and IL-3 to produce cRBCs from hEBs [17]. Bouhassira et al. previously showed that ESCs can differentiate into cRBCs using embryonic and fetal globins [16, 55]. Using a similar protocol to differentiate ESCs from RBCs as the previous study [16, 55, 56], they cocultured transfected iPSCs with FH-B-hTERT to differentiate iPSCs into CD34^+^ HSCs. They then used hydrocortisone, IL-3, BMP4, FLT-3 ligand, SCF, EPO, and IGF1 to amplify and differentiate CD34^+^ cells, leading to erythroid lineage cells with embryonic and fetal globins [56]. Dias et al. used a co-culture system with OP9 stromal cells, and differentiated hESCs and hiPSCs to RBCs expressing predominately embryonic ε and fetal γ globin, and some β globin [57]. Finally, most recently, Douay et al. used SCF, TPO, FLT-3 ligand, BMP4, VEGF, IL-3, IL-6, and EPO, which lead to an enucleation rate of 70 ± 4% in vitro. This technique shows promise for generating fully mature RBCs in vitro [17, 58].

## Technical challenges and breakthroughs

There still remain several hurdles which must be overcome before in vitro-generated RBCs can be widely used for transfusion purposes. Here, we discuss the basic principles and details of enucleation, RBC maturation, and difficulties in large-scale manufacturing, which are major restraints that limit the clinical application of iPSCs. We will also present possible methods to overcome these limitations, and the current progress of each major research group in overcoming these constraints.

## Enucleation

Inadequate enucleation is a major barrier to the large-scale in vitro generation of RBCs, due to the complexity of the enucleation process and the difficulties in recapitulating enucleation ex vivo. Enucleation is a distinct property of native RBCs that is clinically relevant, as nucleated cells do not properly function as RBCs when transfused [59].

**Fig. 1:**
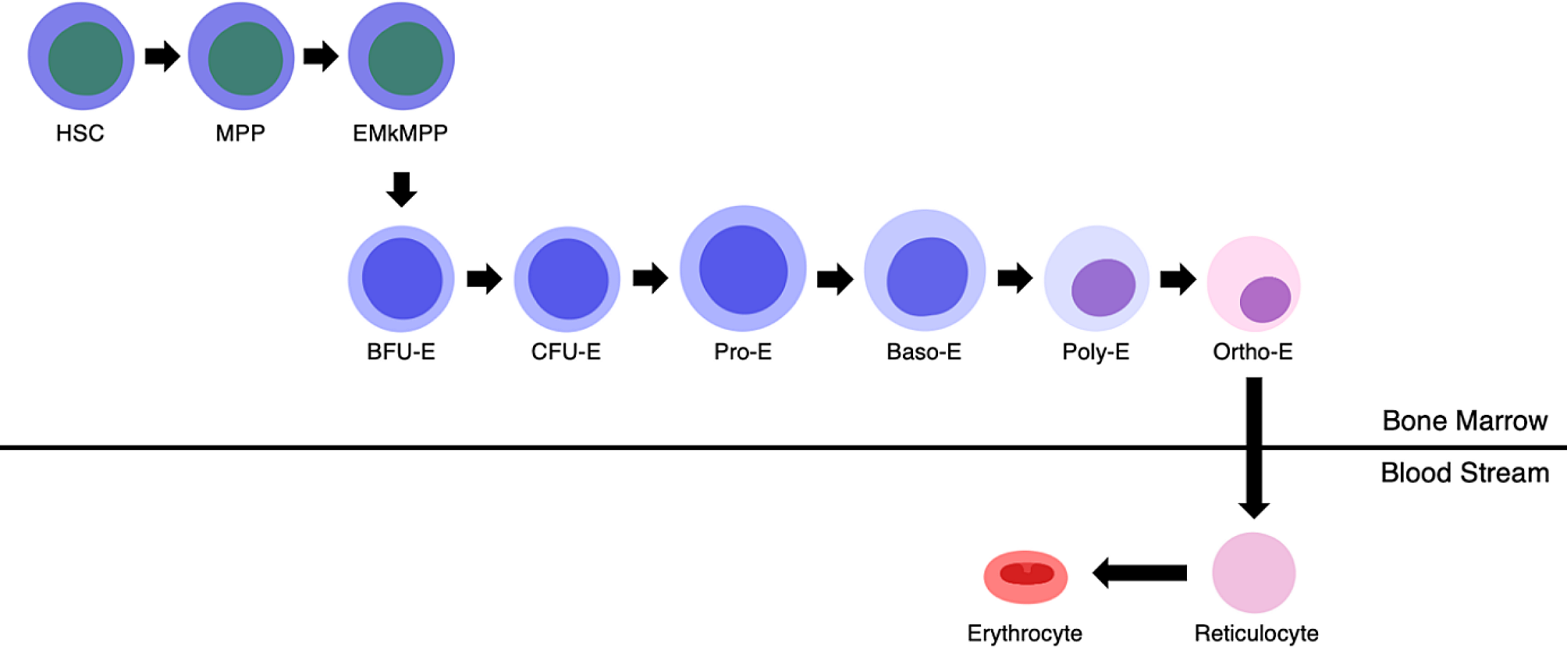
Schematic showing the process of in vivo erythropoiesis. *HSC* hematopoietic stem cell, *MPP* multi-potent progenitor, *EMkMPP* erythroid-megakaryocyte primed multi-potent progenitors, *BFU-E* burst-forming unit erythroid, *CFU-E* colony-forming unit erythroid, *Pro-E* proerythroblast, *Baso-E* basophilic erythroblast, *Poly-E* polychromatic erythroblast, *Ortho-E* orthochromatic erythroblast

Enucleation occurs during the differentiation of Ortho-E into reticulocytes, as their condensed and polarized nuclei exit the cell in the form of pyrenocytes [60]. Nuclear condensation and polarization occur during the conversion of Pro-E to Ortho-E (Fig. 1), and are stimulated by various chromatin factors and histone-modifying proteins [41, 61, 62]. In Ortho-E, the cell cycle is coordinated by cyclin D3 to prepare for enucleation [63, 64]. After these prerequisites are met, Ortho-E expels their nuclei and generates reticulocytes (containing most of their cytoplasm) and pyrenocytes (containing the condensed nuclei) [59, 65]. Enucleation allows for the biconcave shape of RBCs, which increases their deformability for migrating through small capillaries [60, 66]. In addition, the extra space generated by expelling their nuclei allows RBCs to hold increased hemoglobin for carrying oxygen [60, 67]. After pyrenocytes are detached from Ortho-E, phosphatidylserine is expressed on their cellular membrane, which is an apoptotic signal; these pyrenocytes will eventually be phagocytized by EBI macrophages [40, 68]. Reticulocytes will also discard their organelles to increase their hemoglobin content and reduce their size; these organelles are also eaten by resident macrophages [21, 33].

Since high-purity enucleated RBCs are essential for clinical usage, researchers have strived to achieve high enucleation rates. We present a summary of the enucleation rates achieved by different erythrocyte differentiation protocols using various cell sources, including cord blood HSPCs, hESCs, and hiPSCs (Table 1).

As enucleation is affected by various factors and controlled by complicated processes [41, 69], identifying the optimal conditions for achieving high enucleation rates is difficult. The maturation of erythroid lineage cells can be affected by factors such as the cell source used for in vitro erythropoiesis [70], cell culture conditions such as pH [71] and oxygen levels [72], cytokines used in the protocol [73], culture period [69], cell-cell interactions [74, 75], and the aforementioned prerequisites of enucleation [30]. The cell lineage used can significantly affect the enucleation rate when RBCs are generated in vitro. As seen in studies by Giarratana et al. and Miharada et al., cord blood HSPCs can achieve an enucleation rate of 90% when co-cultured with murine MS-5 stromal cells or human mesenchymal stem cells, or 77.5% when cultured in feeder-free conditions [43, 44]. Lapillonne et al. reported that hESCs have an enucleation rate of 52–66%, while hiPSCs have an enucleation rate of 4–10%, indicating that iPSCs yield fewer enucleated cells than ESCs [17]. Although a direct comparison cannot be made due to variations in cytokine composition, culture period, and cell lines used, it can be assumed that HSPCs are able to produce an overall higher proportion of enucleated cells, followed by hESCs.

**Table 1:** A list of erythrocyte differentiation protocols using various cell sources

Year	Cell source	Enucleation rate (in vitro)	Culture methods	Culture periods (days)	Cytokines/chemicals	Fold-increase	Reference	Strengths/weaknesses of each cell source
2002	CB HSPC	4%	Suspension	21	Flt-3 ligand, TPO, SCF, EPO, IGF1	200,000-fold	[15]	**Strengths of CB/PB HSPCs** - Relatively high enucleation rate achieved [43, 76] - HSPCs have natural lineage commitment to RBCs [20]
2005	CB HSPC, PB HSPC	90%	Suspension, cocultured with MS-5 or hMSCs	21	SCF, IL-3, EPO	1,950,000-fold (CB), 122,000-fold (PB)	[43]	
2006	CB HSPC	77.5%	Suspension	20	SCF, EPO, IL-3, VEGF, IGF2	580,000-fold	[44]	**Weaknesses of CB/PB HSPCs** - High enucleation rate achieved through the use of feeder cells [43] - Lower proliferation capacities compared to hESCs and hiPSCs - Difficulties in obtaining CB HSPCs, as they are only available once in a lifetime [77] - Difficulties in obtaining PB HSPCs, as they are unavailable under natural conditions requiring additional steps in order to harvest the HSPCs [77]
2011	PB HSPC	81%	Suspension	18	SCF, IL-3, EPO,	61,500-fold	[47]	
2012	PB HSPC	55–95%	Suspension	24	SCF, IL-3, EPO,	10,000-fold	[76]	
2008	hESC	10 ~ 30% (feeder-free), 30 ~ 65% (OP9)	Suspension, cocultured with OP9, EB formation	42	SCF, EPO, TPO, VEGF, bFGF, Flt-3 ligand, BMP4	10,000-fold	[78]	**Strengths of hESCs** - Expected to have high expansion potential - High proliferation capacities compared to HSPCs
2008	hESC	11%	Monolayer, cocultured with mFLSCs	18	SCF, EPO, TPO, IL-3, IL-6, CSF3	100-fold	[51]	**Weaknesses of hESCs** - Ethical concerns remain [79] - Safety issues in tumorigenicity [80]
2010	hESC, hiPSC	52 ~ 66%(hESC), 4 ~ 10%(hiPSC)	Suspension	26	SCF, EPO, TPO, Flt-3 ligand, BMP4, VEGF, IL-3, IL-6	3500-fold (hESC), 225 ~ 400-fold(hiPSC)	[17]	
2011	hiPSC	2 ~ 10%	Monolayer, cocultured with OP9, mMS-5	60	SCF, EPO, TPO, IL-3, IL-6	10,000-fold	[57]	**Strengths of hiPSCs** - Expected to have high expansion potential - High proliferation capacities compared to HSPCs - Filtration of nucleated cells before the final harvesting can compensate for the low enucleation efficiency [70, 81]
2012	hiPSC	20 ~ 26%	Suspension	52	SCF, EPO, TPO, Flt-3 ligand, BMP4, VEGF, IL-3, IL-6	–	[58]	
2015	hiPSC	21 ~ 29%	Suspension	18	SCF, EPO, TPO, IL-3, IL-6, VEGF, Flt-3 ligand, BMP4	600-fold	[82]	
2015	hiPSC	42 ~ 65% (EB20) 0% (EB9)	Suspension	45 (EB20) 34 (EB9)	SCF, EPO, TPO, FLT-3 ligand, BMP4, VEGF, IL-3, IL-6	–	[69]	
2016	hiPSC	10%	T75 Flask	31	SCF, EPO, TPO, Flt-3 ligand, BMP4, VEGF, IL-3, IL-11, IGF1	200,000-fold	[83]	
2018	hiPSC	28 ~ 40%	Suspension, cocultured with hMSCs	46	SCF, EPO, TPO, Flt-3 ligand, BMP4, VEGF, IL-3, IL-6, bFGF, G-CSF, GM-CSF	10,000-fold	[84]	**Weaknesses of hiPSCs** - Safety issues in tumorigenicity [80] - Low enucleation rate compared to HSPCs [83, 85]
2019	hiPSC	40 ~ 60%	Monolayer, cocultured with HCFC	56	SCF, EPO, IL-3	100 ~ 1000-fold	[86]	
2019	hiPSC	44%(Not filtrated) 99%(Filtrated)	Suspension	45	SCF, EPO, TPO, IBMX, Dex, β-estradiol	100,000-fold	[70]	
2021	hiPSC	6% (No OP9) 18 ~ 60% (OP9)	500mL S. Flask Cocultured with OP9	35	SCF, EPO, TPO, Flt-3 ligand, BMP4, VEGF, bFGF, IL-3, IBMX, β-estradiol	1000-fold	[85]	
2022	hiPSC	90 ~ 99% (Filtrated)	Suspension EB formation	46	SCF, EPO, IL-3	–	[81]	

Under similar conditions, hiPSCs are unable to produce sufficient numbers of enucleated cells [17]. The use of feeder cells is also key in determining the enucleation rate. Lu et al. generated RBCs from hESCs using OP9 feeder cells to provide a suitable microenvironment for cell differentiation and compared their results with those achieved in feeder-free conditions [78]. Feeder-free erythropoiesis resulted in only 10–30% enucleated cells compared to 30–65% in the OP9 co-culture system, suggesting that the co-culture conditions can improve the enucleation rate considerably. Finally, the culture period affects the enucleation of erythroid lineage cells. In a study by Olivier et al., the prolonged PSC robust erythroid differentiation (PSC-RED) protocol achieved higher enucleation than normal length PSC-RED by 50. However, it produced fewer cRBC/iPSCs overall, indicating that the culture period can affect both enucleation rate and erythroid expansion [70]. Rouzbeh et al. generated RBC from differentiated hESCs using embryoid bodies (EBs). They reported that terminal erythroid differentiation was dependent on EB age, as early hEBs from day 9 (EB9) showed no enucleation. In contrast, late hEBs from day 20 (EB20) showed an enucleation rate of 42–65% [69]. Because of the complex interactions governing the enucleation process, further studies on optimal culturing methods, culture periods, and the composition of cytokines and chemicals are required to achieve high enucleation efficiency.

**Fig. 2:**
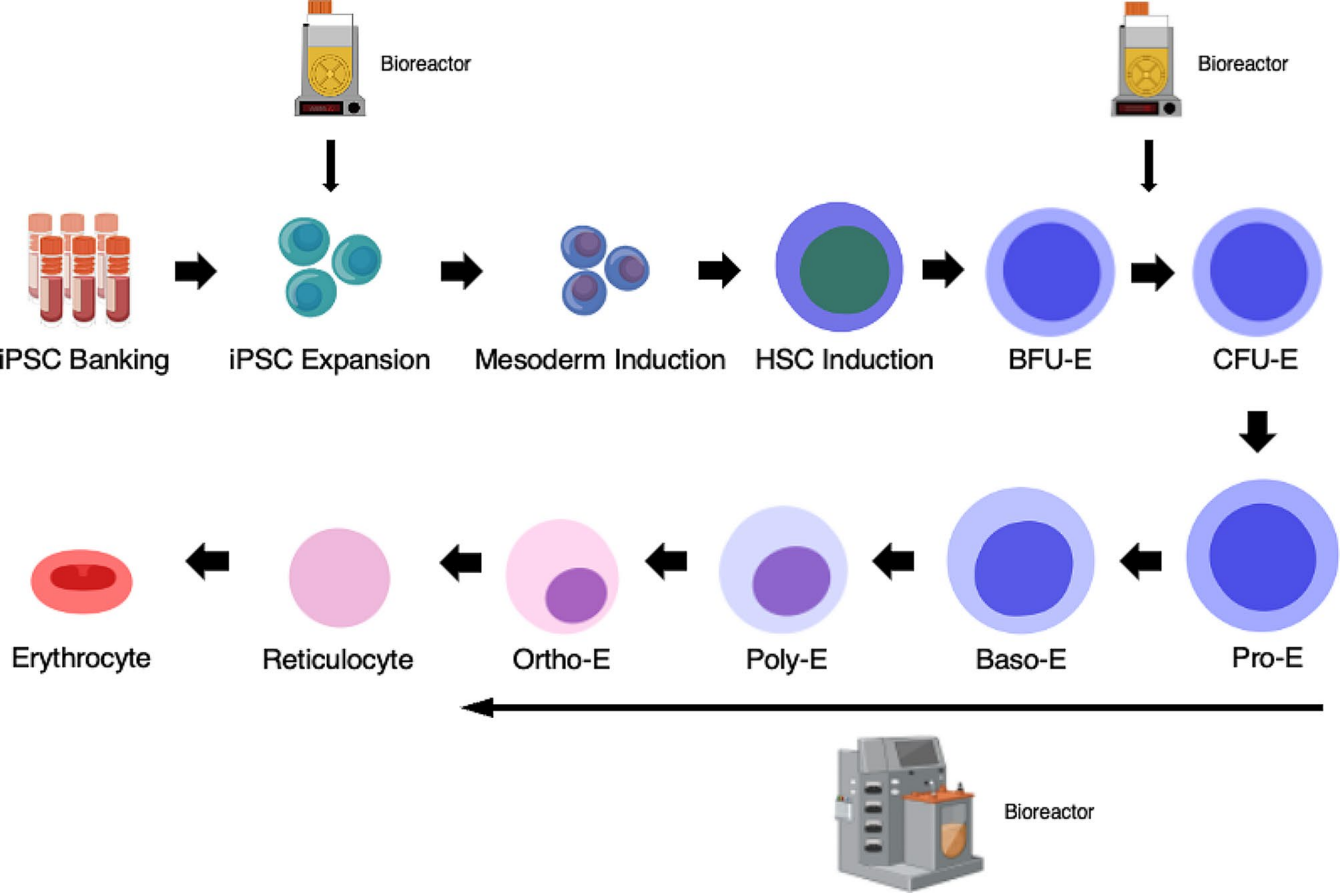
Schematic showing the application of bioreactors in in vitro erythropoiesis

Because identifying novel conditions to achieve high yields of enucleated cells is difficult, filtration has been used as an alternative method to obtain high-purity enucleated cells [87]. Olivier et al. filtered RBCs using a white blood cell (WBC) syringe filter and acquired near 99% enucleation [70]. Bernecker et al. also used filtration with a syringe filter and achieved 94.7%±5.2% enucleation. Although these findings suggest that filtration is a superior method for obtaining high yields of terminally mature and nucleated cells, it has a significant limitation; filtration reduces the total yield of erythroid lineage cells. Since transfusion requires enucleated cells in comparable concentrations to that of standard RBCs, which is around 2 × 10^12^, this is a crucial challenge to overcome [88]. Novel solutions are needed, since neither the enucleation rate nor the number of cells yielded can be compromised. Since progress on improving enucleation yields is beginning to slow down, bioreactors and iPSCs may be the best alternative, as iPSCs have outstanding proliferation ability and bioreactors can amplify the expansion of cells, which can offset the low cellular yield post-filtration (Fig. 2).

### Maturation of red blood cells

There are several standards when determining the maturity of RBCs, including enucleation, hemoglobin (Hb) type, and shape. Mature RBCs have a distinct biconcave shape. Erythroid lineage cells have different types of hemoglobin according to the type of erythropoiesis that they undergo [23]. Hemoglobin is composed of four heme groups and four globin chains, and the globin chains determine its type [89]. Embryonic hemoglobin (HbE) in primitive erythroblasts is created from primitive erythropoiesis, and has two ɑ-globins and two ε-globins (α2ε2), two ζ-globins and two ε-globins (ζ2ε2), or two ζ-globins and two γ-globins (ζ2γ2) [90]. After the erythropoietic niche shifts from the yolk sac to the fetal liver, the transition from primitive erythropoiesis to definitive erythropoiesis has occurred [23]. Hemoglobin produced from definitive erythropoiesis differs from that of produced from primitive erythropoiesis, as definitive erythropoiesis yields cells containing fetal hemoglobin (HbF), with two ɑ-globins and two γ-globins (α2γ2) [90]. After birth, the type of hemoglobin produced switches again, as the erythropoietic niche migrates from the fetal liver to the bone marrow. Here, definitive erythroblasts with adult hemoglobin (HbA) containing two ɑ-globins and two β-globins (α2β2) are generated [91]. In previous reports on the in vitro generation of RBCs, the characteristics of in vitro erythropoiesis were usually similar to those of definitive erythropoiesis in the fetal liver; thus, most of the generated cells possessed HbF and possessed a α2γ2 globin composition [56]. Individuals with no hematological abnormalities, such as sickle cell disease or thalassemia, have mostly HbA (97%) and low levels of HbF (< 1%). Thus, transfusion of RBCs with HbA would likely be more favored [92]. Therefore, finding methods to silence embryonic and fetal globin is necessary in establishing a foundation for transfusing in vitro-generated RBCs. Many studies have sought ways to prevent HbE and HbF production and promote HbA production.

After RBCs are generated, they must be tested to determine whether they are fully mature and possess the distinct features of in vivo-generated RBCs. In addition to the type of hemoglobin, the morphology of the generated RBCs should also be considered. The morphology of erythroid lineage cells can be assessed using the following parameters: shape, size, and color [93, 94]. Flawed RBCs often exhibit different shapes, which can cause poikilocytosis [93]. Native RBCs have a unique biconcave shape [95]. Elliptical RBCs or elliptocytes can indicate an insufficient iron supply during ex vivo erythropoiesis, and were originally seen in patients with iron deficiency anemia; transfusion of these cells may lead to decreased oxygen carrying capacity. Size and color are also parameters to identify mature RBCs, since fully matured RBCs range from 7 to 8 μm and contain an orange-red cytoplasm [28, 96]. Erythroblast precursors range from 8 μm (Ortho-E) to 19 μm (Pro-E), and usually have a grayish cytoplasm [97].

Many other assessments can be performed to evaluate the maturity and condition of RBCs [98]. Quality control is necessary when developing transfusable RBCs [99] and when iPSCs are used for clinical applications [100].

### Difficulties in large-scale manufacturing

To utilize in vitro erythropoiesis-generated RBCs in therapeutics, large-scale manufacturing methods need to be developed to generate RBCs in quantities sufficient for the needs of transfusion purposes, as a single transfusion unit of standard RBCs contains 2 × 10^12^ RBCs [101]. To meet these demands, various protocols have been developed to produce large quantities of RBCs using sources including HSPCs, ESCs, and iPSCs [102–104]. In 2016, Sivalingam et al. developed a serum-free and chemically defined hESC-derived RBC generating protocol using a microcarrier-based suspension culture platform for a promising future scale-up potentiality [103]. Usage of microcarriers on the stage of EB induction have resulted in successful terminal maturation of erythroblasts, in contrast to the failure of erythroblast induction in the control group, where EB induction was conducted through a conventional method. Sivalingam et al. in 2021, developed a protocol to scale up the hiPSC-derived RBC generating process in vitro, utilizing 50mL shake-flasks and 500mL spinner flasks employing the microcarrier-based suspension culture platform in the early phases of the RBC differentiation [85]. They came up with a protocol using the perfusion bioreactor system and achieved maximal cell density of 34.7 million cells/mL, exceeding their previous protocol with maximal cell density of 17 million cells/mL [104]. As a standard unit of blood contains approximately 1–2 × 10^12^ cells, cell densities should exceed 1 × 10^8^ cells/mL to satisfy the requirements, and the usage of bioreactors may be a key to meet the demands [85, 105, 106].

## Conclusions

Blood transfusion is necessary for clinical practice; therefore, the limited supplies of red blood cells (RBCs) must be addressed. Researchers have made progress in the generation of RBCs in vitro using HSPCs, ESCs, and iPSCs using various protocols; however, several issues remain to be solved. We discussed possible methods to solve problems related to RBC enucleation, maturation, and large-scale production. Enucleation still remains a major hurdle to overcome, but filtration has shown promise for increasing the final yield of enucleated RBCs when used in combination with bioreactors. However, most of the studies conducted thus far have followed research-grade standards, whereas clinical applications require clinical- and GMP-grade protocols for safe transfusion. Currently, novel techniques for generating cost-efficient GMP-grade RBCs in vitro are urgently needed.

## Data Availability

All data (or sources thereof) relevant to this study are included in the article, and further inquiries can be directed to the corresponding author.

## Abbreviations

HSPCs: Hematopoietic Stem and Progenitor Cells
ESCs: Embryonic Stem Cells
iPSCs: Induced Pluripotent Stem Cells
RBC: Red Blood Cell
AOCs: Artificial Oxygen Carriers
HBOCs: Hemoglobin-Based Oxygen Carriers
PFCOCs: Perfluorocarbon-Based Oxygen Carriers
HSCs: Hematopoietic Stem Cells
MPPs: Multipotent Progenitors
EMkMPPs: Erythroid Megakaryocyte-Primed Multipotent Progenitors
BFU-E: Burst-Forming Unit Erythroid
CFU-E: Colony-Forming Unit Erythroid
Pro-E: Proerythroblasts
Baso-E: Basophilic Erythroblasts
Poly-E: Polychromatic Erythroblasts
Ortho-E: Orthochromatic Erythroblasts
EBIs: Erythroblastic Islands
IGF1: Insulin-Like Growth Factor 1
VEGF-B: Vascular Endothelial Growth Factor B
ICAM4: Intracellular Adhesion Molecule 4
SCF: Stem Cell Factor
EPO: Erythropoietin
IL-3: Interleukin-3
TPO: Thrombopoietin
cRBCs: Cultured Red Blood Cells
hESCs: Human Embryonic Stem Cells
mFLSCs: Mouse FL-derived Stromal Cells
BMP4: Bone Morphogenetic Protein 4
hEBs: Human Embryoid Bodies
EBs: Embryoid Bodies
WBC: White Blood Cell
Hb: Hemoglobin
HbE: Embryonic Hemoglobin
HbA: Adult Hemoglobin
HbF: Fetal Hemoglobin
